# Startle evoked movement is delayed in older adults: implications for brainstem processing in the elderly

**DOI:** 10.14814/phy2.12025

**Published:** 2014-06-11

**Authors:** Ursina A. Tresch, Eric J. Perreault, Claire F. Honeycutt

**Affiliations:** 1 Institute for Biomechanics, ETH Zurich, Zurich, Switzerland; 2 Sensory Motor Performance Program, Rehabilitation Institute of Chicago, Chicago, Illinois; 3 Department of Biomedical Engineering, Northwestern University, Evanston, Illinois; 4 Department of Physical Medicine and Rehabilitation, Northwestern University, Chicago, Illinois

**Keywords:** Aging, brainstem, startle

## Abstract

Little attention has been given to how age affects the neural processing of movement within the brainstem. Since the brainstem plays a critical role in motor control throughout the whole body, having a clear understanding of deficits in brainstem function could provide important insights into movement deficits in older adults. A unique property of the startle reflex is its ability to involuntarily elicit planned movements, a phenomenon referred to as startReact. The noninvasive startReact response has previously been used to probe both brainstem utilization and motor planning. Our objective was to evaluate deficits in startReact hand extension movements in older adults. We hypothesized that startReact hand extension will be intact but delayed. Electromyography was recorded from the sternocleidomastoid (SCM) muscle to detect startle and the extensor digitorum communis (EDC) to quantify movement onset in both young (24 ± 1) and older adults (70 ± 11). Subjects were exposed to a startling loud sound when prepared to extend their hand. Trials were split into those where a startle did (SCM+) and did not (SCM−) occur. We found that startReact was intact but delayed in older adults. SCM+ onset latencies were faster than SCM− trials in both the populations, however, SCM+ onset latencies were slower in older adults compared to young (Δ = 8 msec). We conclude that the observed age‐related delay in the startReact response most likely arises from central processing delays within the brainstem.

## Abstract (precis)

Our objective was to utilize the noninvasive startReact phenomenon, which is mediated through the brainstem, to gain insight into brainstem processing in older adults. We found that startReact hand extension was intact but delayed in older adults. The observed age‐related delay in the startReact response most likely arises from central processing delays within the brainstem. Our result that the startReact response is delayed in older individuals highlights that movements (e.g., posture, locomotion) and reflexes (e.g., long‐latency stretch reflexes) that are coordinated by the brainstem may have similar deficits in older adults.

## Introduction

Older adults face muscle weakness (Delmonico et al. 2009), slow motor learning (Boyke et al. 2008), sluggish, variable movements (Yan 2000; Yan et al. 2000), and difficulties with balance and gait (Woollacott and Tang 1997) that impair their ability to interact with their environment. The mechanisms driving these changes have been widely evaluated and include atrophy of the cerebral cortex (Salat et al. 2004), losses of gray and white matter (Jernigan et al. 2001; Resnick et al. 2003), axonal atrophy (Misgeld 2011), changes in neuromuscular sensitivity, and neuromuscular junction remodeling (Deschenes 2011). However, little attention has been given to how age affects the neural processing of movement within the brainstem. Literature about age‐related changes in the brainstem is sparse likely since direct neurophysiological experimentation is challenging in the animal and is not ethically possible in humans.

Still, it can be elucidated from reports that the brainstem is impacted by age. Aging is known to influence several brainstem‐mediated functions such as the sleep‐wakefulness cycle (Hut and Van der Zee 2011), cardiovascular reflexes (Vita et al. 1986), the auditory brainstem response (Backoff and Caspary 1994), and saccadic eye movements (Wilson et al. 1993). Indeed, in rats, age‐related cell shrinkage and loss of neurons were reported in the brainstem (Sabel and Stein 1981). Imaging studies in humans provided further evidence that age‐related volume loss in the brainstem does take place (Lawrence and Kuypers 1968b; Luft et al. 1999; Lambert et al. 2013). Still, we are unaware of any study specifically evaluating how these age‐related changes in the brainstem affect the ability to plan and execute movement. Because the brainstem plays a critical role in motor control throughout the whole body (Buford and Davidson 2004; Drew et al. 2004; Deliagina et al. 2008; Musienko et al. 2008; Honeycutt et al. 2009; Sakai et al. 2009; Stapley and Drew 2009; Honeycutt and Nichols 2010; Baker 2011; Soteropoulos et al. 2012), having a clear understanding of deficits in brainstem function could provide important insights into movement deficits in older adults.

The startReact reflex has previously been used as a probe of both brainstem utilization (Valls‐Sole et al. 1999; Queralt et al. 2008; Carlsen et al. 2009; Honeycutt and Perreault 2012; Honeycutt et al. 2013a; Nonnekes et al. 2014) and movement planning (MacKinnon et al. 2007, 2013; Carlsen et al. 2012; Maslovat et al. 2012). The classic startle reflex that occurs in the presence of a startling stimulus, that is a loud sound, results in a quick whole‐body adoption of a protective stance with a characteristic set of muscle activation patterns generalized by flexion of the neck, elbow, and hand (Landis et al. 1939). StartReact movements are not different from voluntarily elicited movements in terms of velocity profiles and target acquisition (Carlsen et al. 2004a) except that they are released 30–40 msec faster. Since the startReact movement is identical to the planned movement, the startReact response has been used as a probe for assessing brainstem contributions to movement planning. Furthermore, startReact movements are linked to the ability to actively resist perturbations of the arm and whole body (Blouin et al. 2006; Ravichandran et al. 2009, 2013; Campbell et al. 2012) indicating that deficits in startReact movements could lead to deficits dealing with environmental perturbations.

Previous literature indicates that the startle reflex originates in the reticular formation of the brainstem (Davis and Gendelman 1977; Davis et al. 1982) and only tasks that utilize these structures for execution are susceptible to startReact (Carlsen et al. 2009; Honeycutt et al. 2013a). Though the cortex does play a modulatory role, startReact is intact in patient populations with cortical (stroke survivors) and corticospinal (hereditary spastic paraplegic patients) damage (Honeycutt and Perreault 2012; Nonnekes et al. 2014). Thus, startReact movements provide a means to non‐invasively evaluate brainstem function and its contributions to movement planning in humans.

Our primary objective was to evaluate deficits in startReact movements in older adults. While it has not been directly studied, it can be elucidated from the literature that brainstem mediation of movement is impacted by age. The classic startle reflex is delayed, but intact, in older adults (Kofler et al. 2001; Ellwanger et al. 2003) indicating that motor processing in the brainstem is likely impaired. Further, despite the brainstem's known importance in motor planning (Buford and Davidson 2004; Sakai et al. 2009), older individuals rely more heavily on the cortex for motor planning than younger individuals. Specifically, longer and larger prefrontal cortical activation occurs in older individuals even during the planning of very simple tasks (Berchicci et al. 2012). This suggests that a restructuring of neural processing may occur to compensate for age‐related changes in brainstem function. Finally, motor tasks which rely heavily on the brainstem such as locomotion (Shik et al. 1968; Mori 1987; McCrea et al. 1995; Pearson 1995) and balance control (Lyalka et al. 2005; Musienko et al. 2008; Honeycutt et al. 2009; Honeycutt and Nichols 2010) are impacted by age (Woollacott et al. 1986; Shkuratova et al. 2004; Monaco et al. 2010; Krasovsky et al. 2013). Importantly, balance control was indicated to include brainstem startle circuits (Nonnekes et al. 2013) implying age‐related deficits in these pathways.

Based upon previous literature, we hypothesized that startReact hand extension is intact (the presence of startle decreases onset latency) but delayed in older adults when compared to younger individuals. We chose hand extension for two important reasons. First, the classic startle reflex results in hand flexion movement; thus by choosing hand extension, we can differentiate movements from the classic startle response allowing a clean method for identifying an intact startReact. Second, reticulospinal connections to the hand are weak and few in number (Riddle et al. 2009); therefore if restructuring of neural processing during movement is present, it would likely first affect those movements that rely less significantly on the reticular formation. A correct hypothesis would indicate that brainstem processing is delayed in older adults, but despite this, the brainstem is still utilized by older adults during motor planning.

## Materials and Methods

### Ethical approval

Data were collected from 20 young subjects (11 males, nine females; age, 24 ± 1) and 24 older subjects (13 males, 11 females; age, 70 ± 11). Subjects were interviewed to ensure that they had no recent upper body injuries (most specifically of the hand, forearm, or neck), hearing sensitivity, or heart conditions. All participants gave informed written consent prior to participating in the study in accordance with the provisions set forth by the Northwestern University Institutional Review Board IRB (STU9204).

### Experimental set‐up

Bipolar electromyography (EMG) electrodes (solid gel, Ag/AgCl surface electrode, MVAP Medical supplies, Inc., Newbury Park, CA) were used to record muscle activity from the extensor digitorum communis (EDC) on the right arm and from the left and right sternocleidomastoid muscle (SCM). For grounding, a unipolar ground electrode (solid gel, Ag/AgCl surface electrode, MVAP Medical Supplies, Inc.) was positioned on the right styloid process. EMG signals were preamplified (Model# AMT‐8; base system, Model# APE‐500; 500 gain Bortec, Calgary, AB, Canada) and filtered with a band‐pass filter of 10–1000 Hz. Next, the signals were anti‐alias filtered by fifth order Bessel filters with a 500 Hz cutoff frequency and sampled at 2500 Hz (PCI‐DAS1602/16; Measurement Computing, Norton, MA).

Subjects were comfortably seated in a chair with armrests supporting hands and arms against gravity. To minimize motion and to ensure a similar position for every subject, participants were strapped across the chest to the chair. Further, the right elbow joint was in a flexed position (90°) and placed in line with the right shoulder to ensure a comparable neutral posture.

Visual feedback was delivered via a monitor placed approximately 3 feet in front of the subject. The screen displayed reaction times (milliseconds) and a visual representation of the relative success of the trial. Specifically, a happy face was displaced to indicate an appropriate onset latency and an unhappy face displayed when subjects initiated movement too slowly. The inclusion of visual feedback was important because the level of arousal and attention is known to improve the size and probability of startle responses (Carlsen et al. 2011).

**Figure 1.**
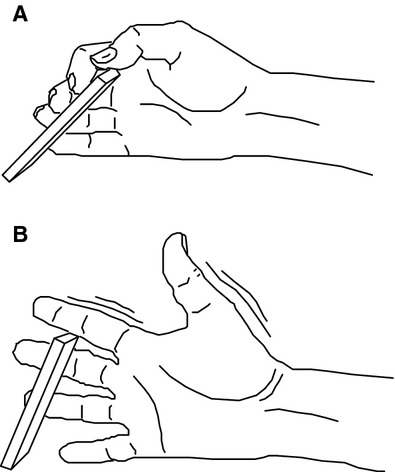
Illustration of the extension task. (A) Hand configuration during the resting position and (B) after completing the extension task.

Subjects were asked to extend their hand from a neutral, resting position with the palm oriented in the sagittal plane and the thumb on the top (Fig. 1A). A switch device was placed such that the switch was pressed when participants were in a resting position and was released when they extended their hand (Fig. 1B). The switch device was used during data processing to ensure that the task was executed correctly.

### Protocol

Subjects were instructed to extend their hand in response to two nonstartling, low‐intensity acoustic sounds (80 dB; low‐intensity trial). The first sound was a WARNING sound, which signaled the subject to get ready and plan the extension task. The second sound was a GO signal, where the subject was asked to perform the extension movement as fast as possible. To avoid anticipation of the GO, the time between the WARNING and GO was randomized between 1.5 and 3.5 sec (Cressman et al. 2006).

Following training, participants performed 4–6 blocks with 15 trials each. During each block, five trials were randomly selected to be either classic startle or high‐intensity trials (i.e., startReact). For classic startle trials, the WARNING signal was replaced with a startling acoustic stimulus (128 dB). In these trials subjects experienced the startling stimulus when they were not prepared to move (classic startle). For high‐intensity trials, the GO signal was replaced by a startling acoustic stimulus. Under these conditions, the subject had already planned the hand extension movement. The startling acoustic stimulus was emitted from a loudspeaker fixed to the chair directly behind the head of the subject. There was no instruction provided on how to react to this sound.

### Data analysis

Onset latency of the EDC and SCM muscles were determined for each trial. The EMG signal was first rectified and filtered with a 10‐point moving average filter. Then, onsets were identified with the help of an automatic detection file in Matlab (R2011b; the MathWorks, Natick, MA), which automatically tagged the time point when the EMG signal rose significantly over background. All trials were then visually inspected for accuracy and to exclude trials when the subjects did not move (the switch device was not altered), moved before the GO signal, or moved too late (muscle activity onset after 400 msec after the Go signal). Trial type and task were blinded to the reviewer.

Next we assessed the presence of a startle during all trial types (low‐intensity, high‐intensity, and classic startle). SCM muscle onset latency within 120 msec after the GO signal indicated a startle occurred (Carlsen et al. 2004a, 2011; Carlsen and MacKinnon 2010). Trials where a startle was detected were designated SCM+, while those without were designated SCM−. Activity in either the right and left SCM muscle was considered (Carlsen et al. 2007; Honeycutt and Perreault 2012; Maslovat et al. 2012).

To determine if startReact was intact, it was first necessary to differentiate between the confounding intensity‐dependent and startle‐dependent effects on reaction time. Faster onset latencies occur in the presence of a startle (Valls‐Sole et al. 1999; startle‐dependent effect) and when the intensity of the GO stimulus is increased (e.g., louder sound; intensity‐dependent effect; Kohfeld 1969, 1971). To identify the intensity‐dependent effect, low‐intensity SCM− trials are compared to high‐intensity SCM− trials. To identify the startle‐dependent effect, high‐intensity SCM+ and high‐intensity SCM− trials are compared to determine if startReact is intact. In addition, we evaluated classic startle trials to confirm reported trends that muscle activity onsets are delayed in the classic startle response in older individuals compared to young (Kofler et al. 2001; Ellwanger et al. 2003). Low‐intensity SCM+ and classic startle SCM− trials were excluded from further analysis as they were elicited only rarely. This procedure resulted in four important trial types: (1) Low‐intensity SCM− trials (LI SCM−); (2) high‐intensity SCM− trials (HI SCM−); (3) high‐intensity SCM+ trials (HI SCM+); and (4) classic startle SCM+ trials (Clst SCM).

Our hypothesis was that startReact would be intact but delayed in older individuals. If a startle‐dependent effect is present, this would indicate that startReact is intact. We further evaluated how age influenced all trial types to determine if a delay was present in older adults compared to young.

Finally, the probability of detecting a startle response (SCM+) was calculated by dividing the number of HI SCM+ and Clst SCM+ by the total number of all high‐intensity trials and classic startle trials, respectively.

### Statistical analysis

Our hypothesis was tested using an analysis of variance (ANOVA) of a linear mixed‐effect model with age (old, young), trial type (LI SCM−, HI SCM+, HI SCM−, Clst SCM+) as the independent factors. Onset latency and probability were considered dependent factors. Subjects were treated as a random effect. Equal variance was not assumed in the statistical model. All individual trials were included in the analysis to decrease the probability of statistical errors by capturing all the variability in the data set (Hedeker 2006). Tukey's Honestly Significant Difference (TukeyHSD), which corrects for multiple comparisons, was applied to compute the pattern of difference between means for all posthoc comparisons. All statistical analyses were computed utilizing R (R Development Core Team, 2008). Differences with a probability lower than 0.05 were considered to be significant. All error bars in figures relate to standard deviations.

## Results

**Figure 2.**
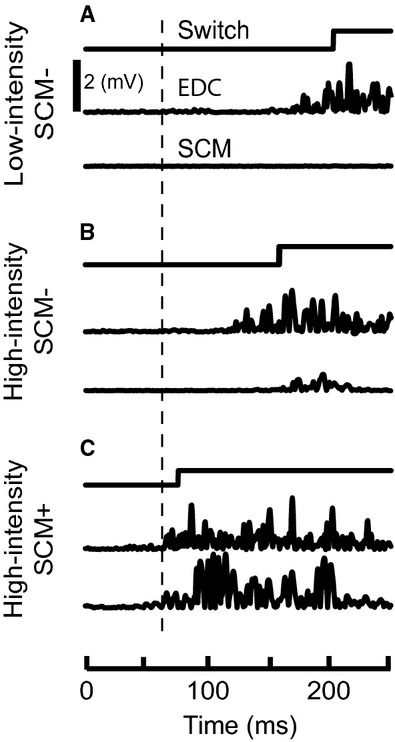
Representative data from hand extension in a young subject. Switch and electromyography (EMG) data from the extensor digitorum communis (EDC) and right sternocleidomastoid (SCM) muscle during high‐intensity SCM+ (A), high‐intensity SCM− (B), and low‐intensity SCM− (C) trials. The thick vertical line is placed to show the onset of EDC activity during high‐intensity SCM+ trials.

Hand extension movements were susceptible to startReact in both young and old subjects as demonstrated by HI SCM+ trials showing faster EDC onset latencies than HI SCM− trials (Figs. 2, 4A, Table 1) EDC muscle onset latency was significantly influenced by trial type (*F*_2,3087_ = 1859.95, *P* < 0.0001) and age (*F*_1,42_ = 5.98, *P* = 0.02). Posthoc comparisons confirmed that the EDC onset latencies were significantly faster during HI SCM+ trials compared to HI SCM− trials for both young and old subjects (*P* ≈ 0). LI SCM− trials were significantly slower than HI SCM+ (*P* ≈ 0) and HI SCM− (*P* ≈ 0) in both young and old subjects.

**Table 1.**
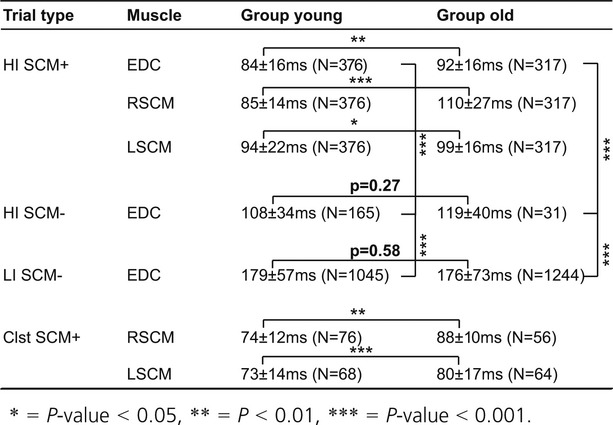
Mean muscle activity onset latencies (msec)

**Figure 3.**
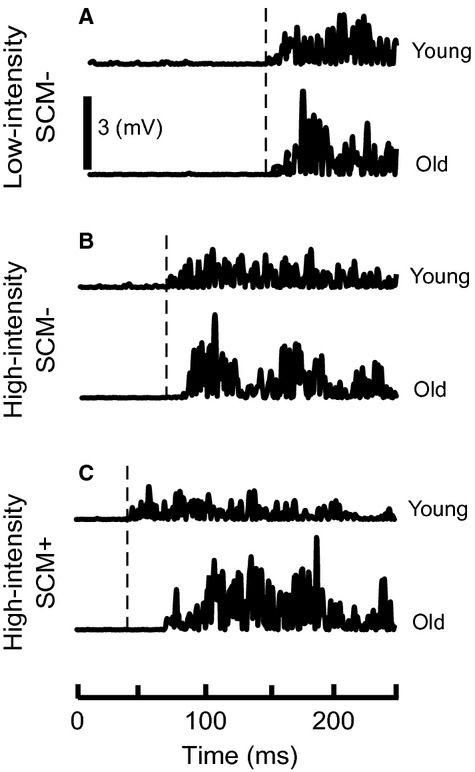
Comparison of muscle activation between young and old subjects. Extensor digitorum communis (EDC) muscle activation during high‐intensity SCM+ (A), high‐intensity SCM− (B), and low‐intensity SCM− (C) trials in young (top) and old (bottom) subjects. Vertical line marks the onset of EDC activity in young subjects.

**Figure 4.**
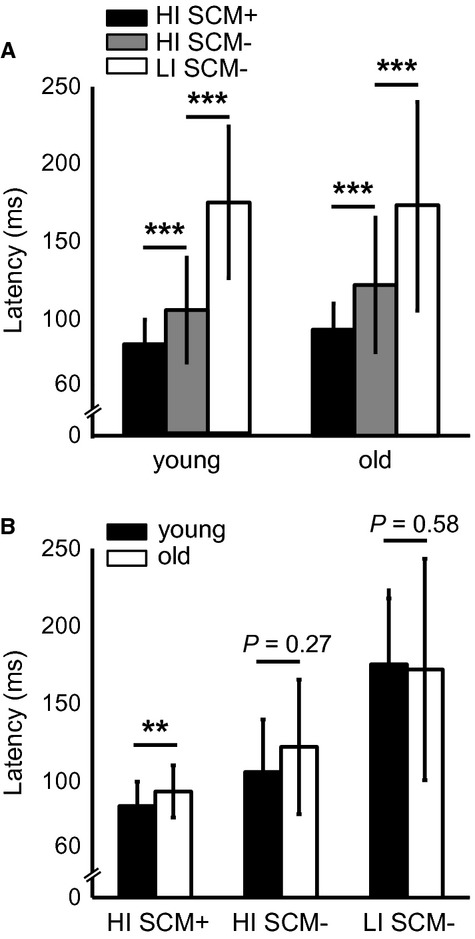
Group statistical results. Comparison of muscle activity onset latencies during high‐intensity SCM+, high‐intensity SCM−, and low‐intensity SCM− trials (A). Comparison of latencies between young and old subjects (B).

Older subjects showed delayed muscle activation during trials when a startle was present (SCM+; Figs. 3, 4B) but not during trials that did not elicit a startle (SCM−). Posthoc comparisons showed that EDC muscle onset latency was significantly delayed in older subjects compared to young during HI SCM+ trials (Δ = 8 msec, *P* = 0.002). Though a delay in HI SCM− trials between young and old was observed, it did not reach significance (Δ = 12 msec, *P* = 0.27) indicating it was not consistent across all subjects. Finally, older subjects were not significantly slower during LI SCM− trials (Δ = 3 msec, *P* = 0.58) suggesting that old and young subjects could perform hand extension equally efficiently.

**Figure 5.**
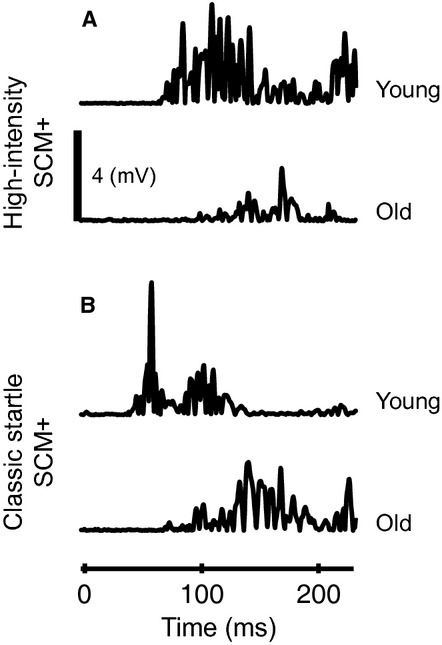
Sternocleidomastoid (SCM) muscle latency comparison between young and old subjects. Right SCM muscle onset latency is depicted for young and old subjects during high‐intensity SCM+ (A) and classic startle SCM+ (B) trials.

**Figure 6.**
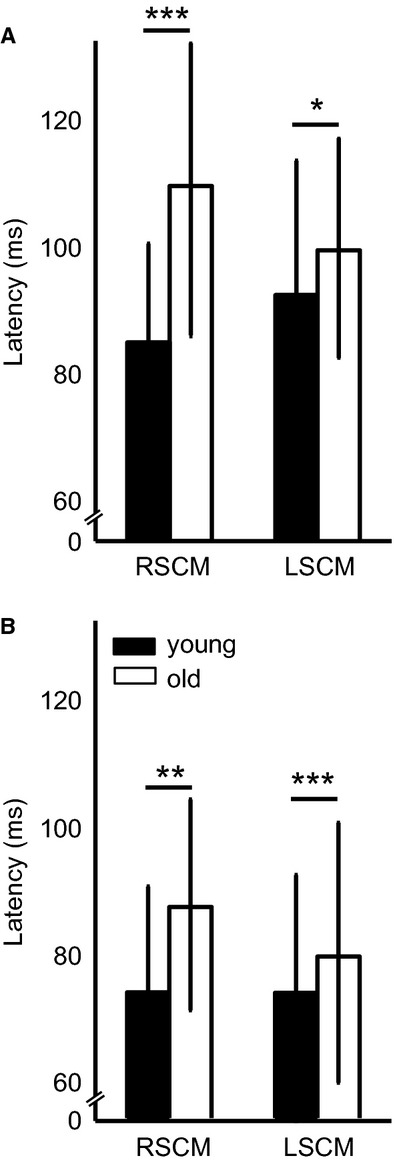
Group results of sternocleidomastoid (SCM) muscle onset latencies in young and old subjects. SCM muscle onset latencies in the right (RSCM) and left (LSCM) in both old and young subjects are compared during high‐intensity SCM+ (A) and classic startle SCM+ (B) trials.

Sternocleidomastoid muscle activation was slower in older adults during both classic startle and high‐intensity SCM+ trials (Figs. 5, 6, Table 1). The right SCM muscle onset latency was significantly influenced by trial type (*F*_1,721_ = 51.07, *P* < 0.0001) and age (*F*_1,41_ = 16.70, *P* = 0.0002). Posthoc comparisons confirmed that the right SCM muscle onset latency was slower in older adults during both classic startle (Δ = 14 msec, *P* = 0.01) and startReact (Δ = 24 msec, *P* ≈ 0) conditions. The left SCM muscle showed a similar, but less robust, trend. Onset latency was influenced by trial type (*F*_1,730_ = 69.00, *P* < 0.0001) though not age (*F*_1,41_ = 2.66, *P* = 0.10). The interaction showed a trend toward significance (*F*_1,730_ = 2.813, *P* = 0.09); therefore posthoc comparisons were completed that demonstrated that the left SCM muscle onset latency was slower in older adults during both classic startle (Δ = 7 msec, *P* = 0.001) and startReact (Δ = 5 msec, *P* = 0.03) conditions.

The onset latency of both right and left SCM muscles were faster during classic startle than during startReact in both young and old adults. The onset of the right SCM muscle was faster during classic startle compared to startReact in both young (Δ = 11 msec, *P* ≈ 0) and old (Δ = 22 msec, *P* ≈ 0) subjects. The left SCM muscle showed the same result in both young (Δ = 22 msec, *P* ≈ 0) and old (Δ = 19 msec, *P* ≈ 0) subjects.

The probability of detecting a SCM+ did not reach statistical significance during high‐intensity trials but was lower during classic startle trials in older adults. The probability of eliciting a SCM+ was influenced by age (*F*_1,42_ = 6.36, *P* = 0.02) but not trial type (*F*_1,40_ = 0.24, *P* = 0.62) with no interaction effect (*F*_1,42_ = 0.11, *P* = 0.73). The probability of eliciting a SCM+ was decreased in older adults during classic startle (old: 0.46 ± 0.35; young: 0.69 ± 0.34; *P* = 0.04), but did not reach statistical significance during high‐intensity trials (old: 0.50 ± 0.34; young: 0.70 ± 0.30; *P* = 0.06) though there was a trend toward significance. There was no difference in the probability of eliciting a SCM+ between classic startle and high‐intensity trials within each population (old: *P* = 0.55; young: *P* = 0.99) indicating that each population's sensitivity to SCM+ was the same during both classic startle and high‐intensity trials.

## Discussion

### Summary

In older subjects, the presence of a startle (SCM+) resulted in faster reaction times during high‐intensity trials compared to high‐intensity trials when startle was not present (SCM−) indicating that older individuals possess an intact startReact response. Therefore, despite evidence that older individuals rely more heavily on the cortex for movement planning (Wild‐Wall et al. 2007; Berchicci et al. 2012) and evidence of brainstem volume reduction (Sabel and Stein 1981; Luft et al. 1999; Lambert et al. 2013), our findings reveal that brainstem structures are likely still involved in motor planning and execution. Still, startReact movements (HI SCM+) were delayed in older adults compared to young indicating that the brainstem processing is likely impacted by age. Importantly, the probability of eliciting a startReact (HI SCM+) and the latency of voluntary (LI SCM−) hand extension movements were not different between young and old individuals suggesting that the observed delay is not related to differences in the ability to execute the task.

### Mechanism driving startReact delay

The observed age‐related delay in the startReact response likely arises from central processing delays within the brainstem. Although the specific neural mechanism remains debated, there are several key papers that indicate that the cortex and corticospinal tract is not the primary neural tract utilized during startReact. First, individuated movements of the hand that are expressed predominately through the corticospinal tract (Lawrence and Kuypers 1968a; Kuypers 1981; Schieber 2004, 2011; Lemon et al. 2012) are not susceptible to startReact (Carlsen et al. 2009; Honeycutt et al. 2013a). Second, startReact is intact in patient populations with cortical (stroke survivors) and corticospinal (hereditary spastic paraplegic patients) damage (Honeycutt and Perreault 2012; Nonnekes et al. 2014) showcasing that degradation to these pathways does not eliminate the startReact response. Finally, the results from this paper and others (Yordanova et al. 2004) demonstrating that voluntary movements are not delayed during simple ballistic tasks in older adults, highlight that when the cortex is utilized for execution a delay is not observed in older adults. Therefore, despite the fact that the cortex and corticospinal tract are known to modulate startReact (Alibiglou and MacKinnon 2012; MacKinnon et al. 2013; Marinovic and Tresilian 2014), we conclude that the cortex is not a likely source of the delay seen in this particular study. Rather, we suggest that the most likely source of the delay is processing delays within the reticular formation. Importantly, we are evaluating a simple reaction time task and this conclusion likely does not extend into more complex tasks that rely more heavily on cortical support. Still, this report indicates that some of the delays observed in those more complex tasks may be due in part to brainstem processing delays.

An alternative mechanism that could explain the observed delay in the startReact and classic startle response is an age‐dependent slowing in central and peripheral motor conduction. However, voluntarily initiated extension movements were not different in latency between old and young indicating that motor conduction is not impaired during voluntary tasks. Further, previous reports indicate that central and peripheral motor conduction deficits in older adults likely can only account for 3–5 msec of delay in distal muscle (Dorfman and Bosley 1979; Claus 1990), shorter than the 9 msec delay in our report. We conclude that while a potential factor, central and peripheral motor conduction is not the most likely cause of the observed delay in startle responses.

### Motor planning in older adults

Our results showing an intact startReact response indicate that older adults still can utilize the brainstem for planning movement – at least in simple ballistic tasks. Movement planning is a global process that requires all layers of the nervous system (Rushworth et al. 2003; Buford and Davidson 2004; Sakai et al. 2009; Stinear et al. 2009) including the brainstem and reticular formation (Buford and Davidson 2004; Sakai et al. 2009). Recent evidence indicates that older individuals rely more heavily on cortical structures for movement planning (Wild‐Wall et al. 2007; Berchicci et al. 2012) possibly the result of age‐related brainstem volume loss (Sabel and Stein 1981; Luft et al. 1999; Lambert et al. 2013). Further, age‐associated shifts from brainstem (automatic) to more cortical (attentional) control have been observed for locomotion and stance (Zwergal et al. 2012). These shifts are accompanied by reports that older adults do not plan the whole movement in advance, relying more on adjustments during movement execution (online corrections) that result in slower, less smooth ballistic aiming arm movements (Yan 2000; Yan et al. 2000).

The startReact reflex has been previously used as a probe of both brainstem utilization (Valls‐Sole et al. 1999; Queralt et al. 2008; Carlsen et al. 2009; Honeycutt and Perreault 2012; Honeycutt et al. 2013a) and movement planning (MacKinnon et al. 2007, 2013; Carlsen et al. 2012; Maslovat et al. 2012). Only movements that utilize the brainstem (specifically the reticular formation) for movement planning are susceptible to startReact (Carlsen et al. 2009; Honeycutt et al. 2013a). Additionally, startReact is only triggered when a subject is in a state of movement planning. Specifically, if planning is not possible, for example, during a choice reaction time task where the task is not revealed until the GO, a startling stimulus does not result in the early release of movement (Carlsen et al. 2004b, 2008). Our result that hand extension is susceptible to startReact in older individuals indicates that despite probable brainstem processing delays, they still utilize the brainstem for planning movement – at least in the simple tasks described in this report.

### Startle versus StartReact

The results of this paper highlight that the classic startle reflex and the startReact response are closely linked but still appear to be somewhat distinctive phenomena. It has been previously suggested in literature that startle and startReact are unique phenomena (Kumru et al. 2006; Alibiglou and MacKinnon 2012; Honeycutt and Perreault 2012). Our result that the onset latency of SCM activity during the classic startle response was faster than that elicited during startReact responses further indicates that these phenomena are distinctive. While this paper cannot specifically identify why the delay during startReact is present, we suggest that this result could be an artifact of the time differences when the classic startle and startReact trials were collected. Specifically, classic startle responses adapt readily and are only present for a few trials while startReact can be elicited as long as the subject is focused on the experiment. As the earliest classic startle and startReact trials represent the strongest responses, this may explain the change in latency. Alternatively, it could be argued that the startReact response utilizes more complex neural circuitry, and therefore introduces a delay. Thus, despite the fact that startle and startReact are always expressed together and both show delays in older adults, indicating that these phenomena are fundamentally linked, there remains a distinctive quality to these responses.

### Clinical significance

Our result that the startReact response is delayed in older individuals highlights that movements and reflexes that are coordinated by the brainstem may have deficits in older adults. Since the brainstem is known to be highly involved in postural control and locomotion (Lyalka et al. 2005; Musienko et al. 2008; Honeycutt et al. 2009; Honeycutt and Nichols 2010) and the startle reflex has been linked to whole‐body postural responses, (MacKinnon et al. 2007; Queralt et al. 2008; Nonnekes et al. 2013) our result shows that aging delays startle circuits could have implications for the poor postural control seen with age. Furthermore, it has been linked to the ability to react and withstand environmental perturbations (Blouin et al. 2006; Ravichandran et al. 2009, 2013; Campbell et al. 2012). It is known that older adults are less stable and prone to falls (Campbell et al. 1981; Lord et al. 1994). Our results indicate that one potential mechanism may be a delay in brainstem processing limiting older individual's ability to quickly adjust to their environment.

This is the first report to demonstrate that hand extension is susceptible to startReact. It was recently demonstrated that startReact elbow movements were improved in terms of onset latency and muscle activation patterns in stroke survivors compared to voluntary initiated movements (Honeycutt and Perreault 2012). Therefore, if startReact hand extension is present in stroke survivors it may be an attractive therapeutic target addressing the most prevalent impairment following stroke (Latham 1989). Our own preliminary results demonstrate that hand extension is indeed susceptible to startReact in stroke survivors (Honeycutt et al. 2014). Still, further research in stroke survivors is necessary to evaluate if the startReact phenomenon is an appropriate mechanism to enhance hand control.

## Acknowledgments

The authors would like to thank T. Goetz‐Haswell his technical and scientific expertise.

## Conflict of Interest

The authors have nothing to disclose.
