# Erratum

**DOI:** 10.14814/phy2.13230

**Published:** 2017-03-22

doi: 10.14814/phy2.12025

In (Tresch et al. 2014), the following error was published in the Mechanism driving startReact delay subsection.

The Marinovic citation is presented in the final version as:

Marinovic and Tresilian 2014

This is incorrect and should instead be:

Marinovic et al. 2014

The same citation is also left out in the References list.

We apologize for the error.
